# Early changes in photopic negative response in eyes with glaucoma with and without choroidal detachment after filtration surgery

**DOI:** 10.1136/bjophthalmol-2021-320730

**Published:** 2022-04-08

**Authors:** Yuro Igawa, Takuhei Shoji, Robert Weinreb, Yozo Miyake, Yuji Yoshikawa, Shunichiro Takano, Kei Shinoda

**Affiliations:** 1 Department of Ophthalmology, Saitama Medical University, Iruma, Saitama, Japan; 2 Hamilton Glaucoma Center and Shiley Eye Institute, Viterbi Family Department of Ophthalmology, University of California, San Diego, California, USA; 3 Kobe City Eye Hospital, Kobe, Japan; 4 Ophthalmology, Saitama Medical University Hospital, Moroyama-machi, Saitama, Japan; 5 Ophthalmology, Teikyo University School of Medicine Graduate School of Medicine, Itabashi-ku, Japan

**Keywords:** Glaucoma, Electrophysiology, Treatment Surgery

## Abstract

**Background/aims:**

To evaluate the electroretinographic (ERG) changes in the early postoperative period following glaucoma filtration surgery, and its relationship with choroidal detachment (CD).

**Methods:**

This retrospective observational single-centre study included 57 consecutive patients with primary open-angle glaucoma who underwent unilateral glaucoma filtration surgery. The patients were divided into two groups according to the presence or absence of CD. ERG components, including the photopic negative response (PhNR), a-wave and b-wave were compared before and after surgery using skin electrodes.

**Results:**

There were 46 patients in the non-CD group and 11 in the CD group. ERG was recorded within 5.1 (2.1 to 8.1) (mean (95% CI)) days after surgery. In the non-CD group, the PhNR amplitude, PhNR/b-wave amplitude ratio and PhNR implicit time improved significantly after surgery (p=0.008, 0.002 and 0.039, respectively). In the CD group, the amplitude of the PhNR, a-wave and b-wave were significantly deteriorated after surgery (p=0.002, 0.001 and 0.001, respectively). Postoperative intraocular pressure (IOP) (p=0.031) and postoperative CD (p<0.001) were significantly associated with change in the PhNR amplitude in the univariate models. In the multivariate analysis, severe CD (stage 3) cases tended to be deteriorated more.

**Conclusion:**

Even in the early postoperative period within several days, the PhNR amplitude increased with IOP lowering following filtration surgery in the absence of CD. The presence of CD may arrest the improvement of the retinal ganglion cell function. The present results enhance understanding the structural and functional recovery after glaucoma surgery and the role of postoperative CD.

**Key Message**

**What is already known on this topic**

- Electrophysiological assessment of eyes with choroidal detachment, a common postoperative change of glaucoma surgery, has not been reported previously.

**What this study adds**

- In the absence of choroidal detachment, rapid functional improvement was observed in the first, second and third order retinal neurons within several days of glaucoma filtration surgery.

**How this study might affect research, practice or policy**

- The results of this study enhance understanding the structural and functional recovery after glaucoma surgery and the role of postoperative choroidal detachment.

## Introduction

Glaucoma, an irreversible disease, is characterised by the loss of retinal ganglion cells (RGCs) and their axons in the retina, with progressive optic-nerve damage and characteristic visual-field defects.^1 2^ It is the second most common cause of preventable blindness in the world.^3^ In 2020, 3.6 million people over the age of 50 worldwide lost their vision because of glaucoma.^4^ Visual-field loss typically becomes detectable only after a large number of RGCs are lost.^5^ RGC damage can be detected by measuring retinal nerve fibre layer (RNFL) thickness using optical coherence tomography (OCT) to capture morphological changes in the early stages of glaucoma.^6^ The reversibility of some glaucoma-related changes, such as optic disc cupping, lamina cribrosa displacement,^7^ vessel density and ocular blood flow,^8^ following intraocular pressure (IOP) reduction in patients with glaucoma has been reported.

The photopic negative response (PhNR), an electroretinographic (ERG) component, is an objective parameter that can be used to estimate RGC function.^8 9^ It consists of a slow negative wave that follows the positive b-wave of the ERG and is derived from the inner retinal layers, specifically the RGC layer.^8^ The PhNR amplitude and PhNR/b-wave ratio, defined as PhNR divided by the b-wave, have been reported to worsen in glaucoma.^8 9^

Investigations of the function of RGCs^8–10^ as well as of their microstructure^6 11 12^ have contributed to the understanding and diagnosis of the pathophysiology of glaucoma. Interestingly, several studies have shown that the PhNR amplitude is significantly lower in glaucomatous eyes than in normal eyes.^8 9^ Niyadurupola *et al* 10 and Tang *et al* 13 reported that lowering the IOP led to electrophysiological RGC improvement. These studies reported improvements in PhNR in ocular hypertension and glaucoma after several months of IOP-lowering treatments, including eye-drops, laser therapy and surgery. However, there is no information on how early this functional RGC improvement occurs after IOP reduction following glaucoma filtration surgery. Further, there has been no electrophysiological assessment of eyes that developed choroidal detachment (CD), a common postoperative change of glaucoma surgery.^14 15^

In this study, we evaluated RGC function in the early postoperative period in glaucomatous eyes undergoing filtration surgery using full-field ERG and skin electrodes. Further, we compared these changes in eyes with and without CD.

## Methods

### Study enrolment and participants

Patients who underwent glaucoma filtration surgery and preoperative and postoperative ERG recordings at Saitama Medical University Hospital between September 2020 and June 2021 were included. All patients underwent a comprehensive pre-and postoperative ophthalmologic examination, including visual acuity testing, a slit-lamp biomicroscopy and IOP measurement with Goldman applanation tonometry. The most recent preoperative values were used to assess visual acuity. Visual-field tests were performed within 3 months preoperatively. Standard automated perimetry was performed with the Humphrey Field Analyzer (Carl Zeiss Meditec, Jena, Germany) using the 24-2 Swedish Interactive Thresholding Algorithm standard threshold. We measured the axial length (AL) and central corneal thickness (CCT) (Optical Biometer OA-2000, Tomey, Nagoya, Japan) within 3 months preoperatively. All participants underwent cross-sectional imaging to measure the circumpapillary RNFL thickness at each visit using spectral domain OCT (Spectralis OCT, Heidelberg Engineering, Heidelberg, Germany).

Glaucoma was diagnosed based on: (1) glaucomatous changes in the optic nerve head (ONH) observed with fundus photography, such as a vertical cup-to-disc ratio ≥0.7, rim notch with a rim width ≤0.1 and/or an RNFL defect (with its edge at the ONH margin greater than that at a major retinal vessel) diverging in an arcuate or wedge shape; (2) glaucomatous visual field defects that met at least one of the Anderson-Patella criteria, that is, a cluster of ≥3 points in the pattern deviation plot in a single hemifield (superior/inferior) with p<0.05, one of which must have been p<0.01; a glaucoma hemifield test result outside the normal limits, or an abnormal pattern deviation with p<0.05.^16^ The included patients had manifest glaucoma deemed to require glaucoma surgery owing to high IOP or evidence of progression in the visual field. All glaucoma subtypes and treatment modalities were included. Patients with visual acuity ≥20/200 were included in the study, whereas those with diabetic retinopathy, and insufficient ERG quality (described in detail below) were excluded. No exclusion criteria were applied for AL, refractive errors, CCT or past ocular surgery history if the patients fulfilled the inclusion criteria. The patients were divided into two groups according to the presence or absence of CD after glaucoma filtration surgery. The presence of CD and CD grading were determined using ultra-widefield fundus photography (California, Nikon, Tokyo, Japan) and grading criteria as previously reported.^17^

### Full-field ERG recording

Full-field ERG was recorded using the RETeval system (LKC Technologies, Gaithersburg, MD; Welch Allyn, Skaneateles Falls, New York, USA), a portable ERG device that uses skin electrodes. The pupils were dilated with topical 0.5% tropicamide and 0.5% phenylephrine hydrochloride. The patient adapted to the background light prior to testing. Sensor strips of skin electrodes were carefully placed 2 mm below the lower eyelid margin after cleaning the skin with an 80% ethanol-impregnated solution and connected to a lead wire. The stimuli consisted of a red flashing light (intensity of 1.0 cd-s/m^2^, stimulus duration of 4 ms) on a stable blue background light (10 cd/m^2^). Two hundred flashes were delivered at a frequency of 3.4 Hz, which has been reported to achieve a good balance between testing time and signal quality.^18^ Signal acquisition was performed at a sampling frequency of 2 kHz.

The recording time was 220 ms, including 100 ms of prestimulus recordings. The implicit times and amplitudes of the ERGs were automatically analysed using the software integrated into the RETeval system. The a-wave amplitude was measured from the average pre-stimulus mean baseline to the a-wave trough. The b-wave amplitude was measured from the a-wave trough to the b-wave peak; the a-wave and b-wave peak times were measured from the time of the flash to the peak of the wave.^19^ The PhNR was selected as the most negative trough appearing behind the b-wave according to the standard of the International Society for Clinical Electrophysiology of Vision.^8^ Its amplitude can be measured in various ways; in this study, it was measured from the b-wave peak to the PhNR trough (PT) (as shown in online supplemental figure 1). We also analysed the PT/b-wave amplitude ratio; the PT amplitude and PT/b-wave amplitude ratio have been reported to be highly reproducible.^20^ These indices were analysed using the well-recorded ERG waves that had a stable recorded baseline. When the last point of the recorded waveform deviated from the baseline level before recording by 3SD or more of the noise amplitude, it was judged that the baseline of the recorded waveform was unstable and defined as an ERG wave with insufficient quality. The fluctuation range of the baseline before recording was regarded as the noise amplitude. It was measured in 10 randomly selected eyes according to the manufacturer’s instructions and was measured to be 1.3±0.9 µV. Therefore, the reference value was defined as 5.1 µV. Preoperative ERGs were recorded the day before surgery, and postoperative ERGs were measured within 2 weeks.

10.1136/bjophthalmol-2021-320730.supp1 Supplementary data

### Statistical analysis

The significance of the differences within the groups was compared using the paired t-test and that between the groups was compared using Student’s t-test. Pearson χ^2^ and Fisher’s exact test were used for categorical variables. We analysed the relationship between the change in PhNR amplitude and various structural and functional factors such as age, AL, CCT, preoperative and postoperative IOP, preoperative mean deviation (MD) values by HFA 24-2, past surgical history, presence or absence of postoperative CD, change in visual acuity and self-reported systemic diseases. Decimal visual acuity was converted to logarithm of the minimum angle of resolution (logMAR) for statistical analysis. Variables with p<0.10 in the univariate analysis were used for multivariate analysis. In addition, to confirm the intersession reproducibility, we randomly selected 15 patients and measured the preoperative and postoperative PhNR amplitudes and implicit times in the non-operated eye and calculated the coefficient of variation (CV) values. Statistical significance was set at p<0.05 based on a threshold two tailed. Distributed variables are reported as mean (95% CI), except for age, which is reported as the median (quartile). We used the JMP Pro V.16 software (SAS Institute) for the analyses.

## Results

### Participant characteristics

**Figure 1:**
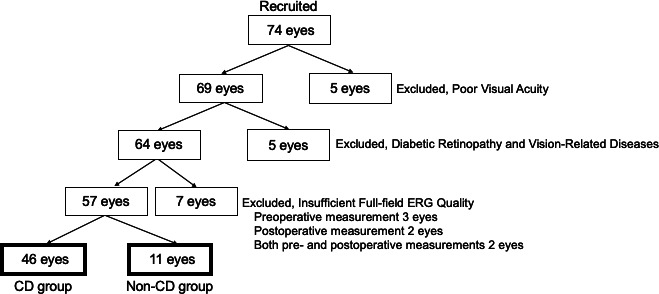
Flow diagram of study patients. CD, choroidal detachment; ERG, electroretinography.

Figure 1 shows a flow diagram of the study patients. Seventy-four patients were initially enrolled in the study. Seventeen patients were excluded because of poor visual acuity (five eyes), diabetic retinopathy (five eyes) and insufficient ERG quality (seven eyes; three eyes had insufficient quality preoperatively, two eyes had insufficient quality postoperatively and two eyes met the criteria for insufficient quality in both preoperative and postoperative measurements). Among the 4 eyes that showed insufficient ERG quality and IOP of less than 10 mm Hg, 2 eyes showed CD (2 eyes out of 11 eyes: 18.2%), and the other 2 eyes had no CD (2 eyes out of 46 eyes: 4.3%), and there was no significant difference. Thus, the data of 57 patients were included in the analysis, including those of 46 patients without CD and 11 with CD. Table 1 summarises the characteristics of the two groups. There were no significant between-group differences in age, gender distribution, preoperative best-corrected visual acuity, preoperative mean deviation, preoperative IOP, distribution of glaucoma subtypes and whether cataract surgery was concomitantly performed. As expected, the postoperative IOP value was significantly lower in the CD group (6.4 (4.6 to 8.1) mm Hg, mean (95% CI)) than in the non-CD group (9.7 (8.6 to 10.7)) mm Hg (p=0.003). Other factors such as age, gender distribution, preoperative IOP, preoperative MD value, CCT, AL, self-reported systemic diseases and past ocular surgery history were not significantly different between the groups.

**Table 1:** Patientcharacteristics

Variables	Non-CD group	CD group	P value
Patients, eyes	46	11	
Gender (female/male), n (%)	19 (41.3)/27 (58.7)	3 (27.3)/8 (72.7)	0.502
Age, mean (quartile), years	70.5 (63.3, 79.0)	73.0 (72.0, 76.5)	0.287
Preoperative BCVA, mean (95% CI), log MAR	0.13 (0.05 to 0.21)	0.29 (0.03 to 0.55)	0.197
IOP, mean (95% CI), mm Hg			
Preoperative IOP	19.3 (17.5 to 21.1)	20.1 (15.6 to 24.6)	0.729
Postoperative IOP	9.7 (8.6 to 10.7)	6.4 (4.6 to 8.1)	0.003*
Preoperative MD (24-2), mean (95% CI), dB	−19.7 (−22.2 to −17.3)	−18.4 (−24.0 to −12.7)	0.627
Preoperative RNFL thickness, mean (95% CI), µm	49.5 (45.3 to 53.6)	53.9 (40.3 to 67.5)	0.494
Periods between surgery and ERG recordings (mean±SD (range)), days	4.9±2.7 (1 to 11)	5.8±3.5 (2 to 14)	0.429
Glaucoma subtype, n (%)			
POAG	39 (84.8)	7 (63.6)	0.207
NTG	2 (4.3)	2 (18.2)	
PACG	1 (2.2)	0 (0)	
PXFG	3 (6.5)	2 (18.2)	
Unidentified	1 (2.2)	0 (0)	
Type of glaucoma surgery, n (%)			
Trabeculectomy	25 (54.3)	7 (63.6)	0.739
Ex-press	21 (45.7)	4 (36.4)	
Glaucoma surgery combined with or without cataract surgery, n (%)			
Glaucoma surgery alone	21 (45.7)	3 (27.3)	0.326
Combined surgery	25 (54.3)	8 (72.7)	
Central corneal tickness, µm	513 (508 to 518)	516 (479 to 552)	0.767
Axial length, mm	25.1 (24.6 to 25.7)	24.6 (23.6 to 25.6)	0.405
Past cataract surgery history, n (%)	10 (22.2)	3 (27.2)	0.700
Past glaucoma surgical history, n (%)	4 (8.7)	1 (9.0)	1.000
Past vitrectomy history, n (%)	2 (4.3)	0 (0)	1.000
Past cerebrovascular event, n (%)	3 (6.5)	0 (0)	1.000
Self-reported hypertension, n (%)	14 (30.4)	5 (45.5)	0.478
Self-reported diabetes, n (%)	8 (17.4)	4 (36.4)	0.196
Change in IOP, mm Hg	9.6 (7.5 to 11.7)	13.7 (9.3 to 18.2)	0.088
Choroidal detachment grading, n (%)			
Grade 1	n.a.	2 (18.2)	
Grade 2		5 (45.5)	
Grade 3		4 (36.4)	

Between-group comparisons were performed with Student’s t-test for all parameters. Fisher’s exact test was only used for glaucoma subtype and surgery. Data are presented as mean (95% CI), except for age, which is presented as the median (quartile).

*P<0.05 was considered significant.

BCVA, best-corrected visual acuity; CD, choroidal detachment; ERG, electroretinography; IOP, intraocular pressure; logMAR, logarithm of the minimum angle of resolution; MD, mean deviation; n.a, not available; NTG, normal tension glaucoma; PACG, primary angle-closure glaucoma; PEA, phacoemulsification and aspiration; POAG, primary open angle glaucoma; PXFG, pseudo-exfoliation glaucoma; RNFL, retinal nerve fibre layer.

### Representative cases

**Figure 2:**
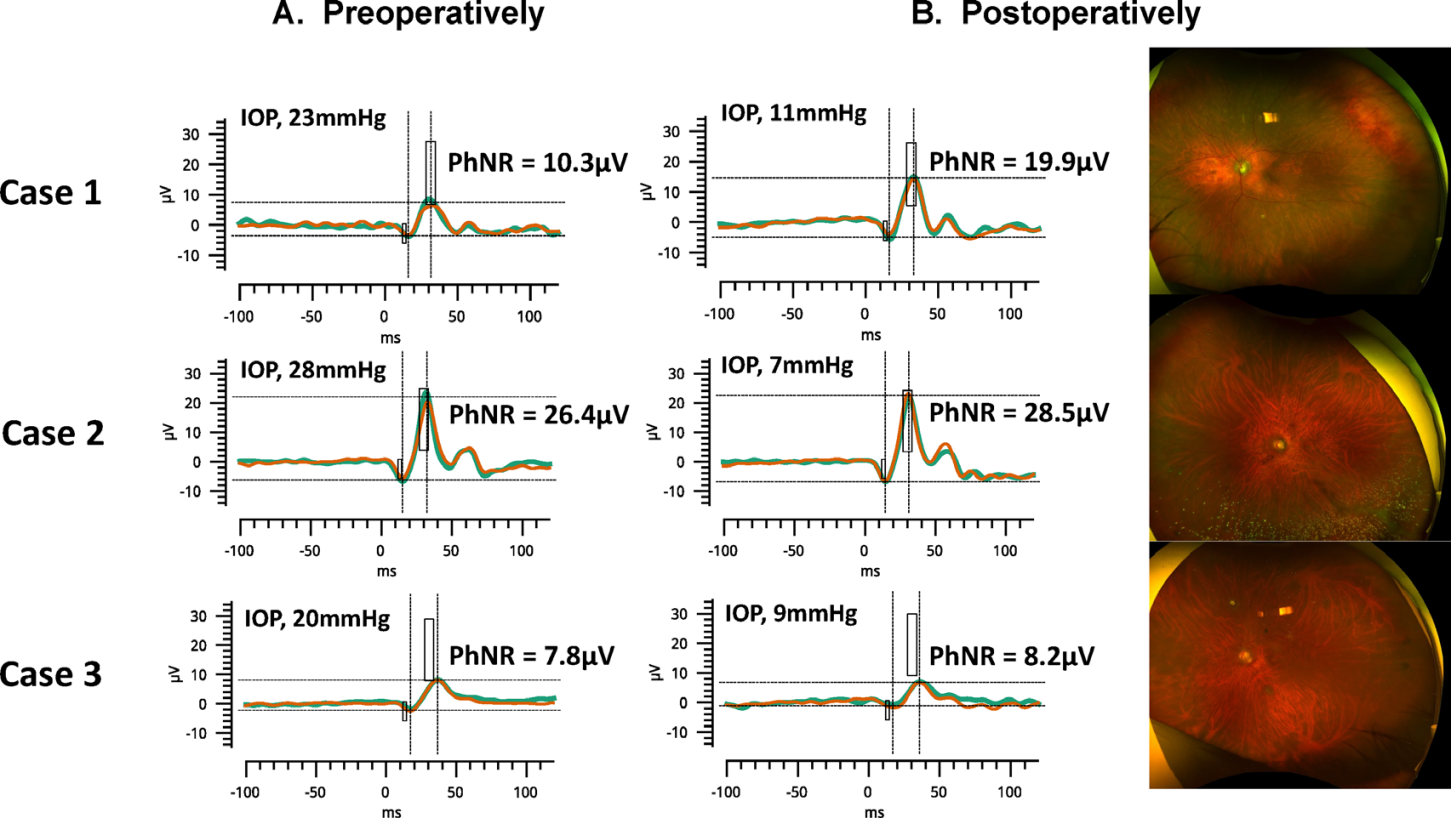
Representative cases from the non-CD group of preoperative (left column) and postoperative (middle column) electroretinography results and widefield fundus photography (right column). Case 1 was an 84-year-old man. His IOP was 23 mm Hg preoperatively. The day after the surgery, his IOP decreased to 11 mm Hg and his PhNR amplitude improved. Case 2 was a 64-year-old man. His IOP was 28 mm Hg preoperatively, and on the seventh day of surgery, his IOP decreased to 7 mm Hg and his PhNR amplitude improved slightly. Case 3 was a 72-year-old man. His IOP was 20 mm Hg preoperatively, and on the third day of surgery, his IOP decreased to 9 mm Hg and his PhNR amplitude improved slightly. CD, choroidal detachment; IOP, intraocular pressure; PhNR, photopic negative response.

**Figure 3:**
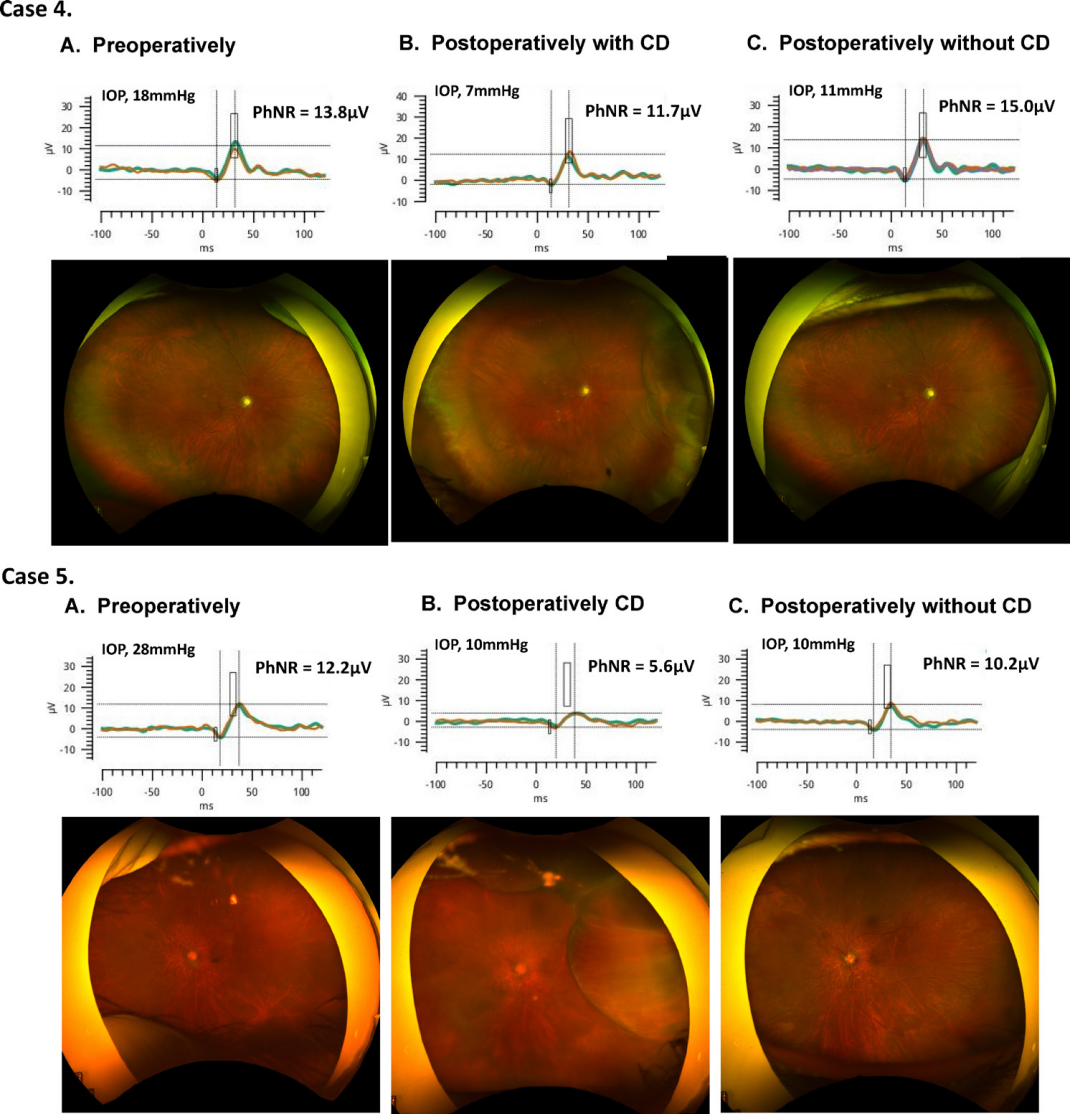
Representative cases of the CD group preoperatively (left column), early postoperatively with CD (middle column), and postoperatively after spontaneous recovery of CD (right column). Case 4 was a 74-year-old woman. Six days after surgery, the IOP decreased to 7 mm Hg, and a grade 2 CD was confirmed by wide-angle fundus photography. PhNR amplitude had also worsened. One month later, the CD recovered spontaneously and the PhNR amplitude improved. Case 5 was a 73-year-old woman. The preoperative IOP was 28 mm Hg. On postoperative day 4, the IOP was 10 mm Hg, but wide-angle fundus photography showed grade 2 CD, and the amplitude of the PhNR also deteriorated. One month later, the CD recovered spontaneously and the amplitude of the PhNR improved. CD, choroidal detachment; IOP, intraocular pressure; PhNR, photopic negative response.

Figure 2 shows three eyes of the representative cases from the non-CD group. Compared with before glaucoma surgery, IOP decreased and PhNR amplitude improved after surgery in all three cases. Figure 3 shows two eyes of representative cases from the CD group. In both cases, transient CD (grade 2) occurred after glaucoma surgery, and PhNR amplitude deteriorated compared with before surgery. CD recovered spontaneously and disappeared after 1 month and the PhNR amplitude also improved.

### Comparison of ERG parameters preoperatively and postoperatively

**Figure 4:**
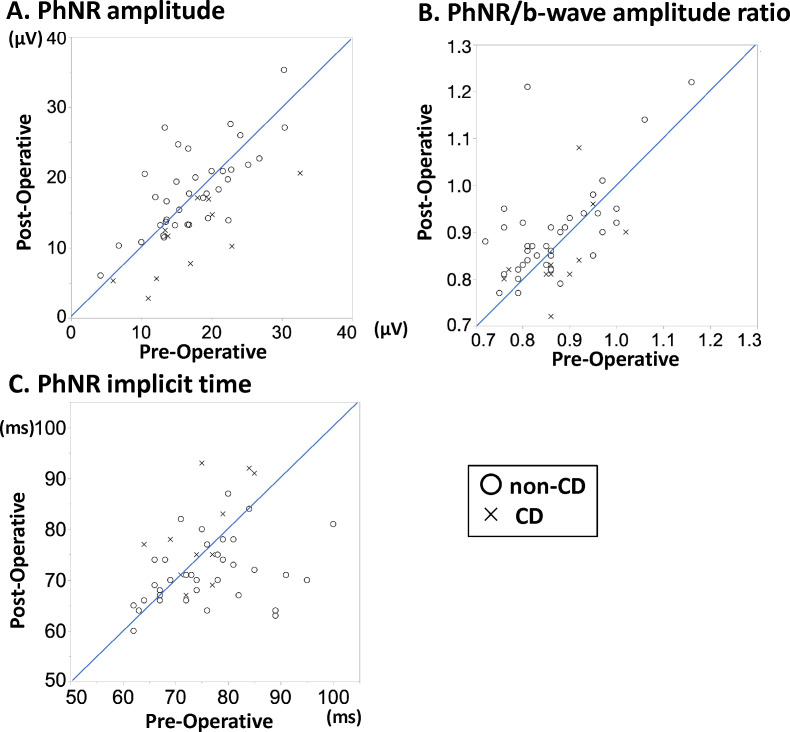
Scatter plots showing the change in PhNR implicit time and amplitude and the PhNR/b-wave amplitude ratio. Scatter plots of each PhNR parameter pre -and postoperatively. The x-axis shows the preoperative values, and the y-axis shows the postoperative values. In the plot, the circle indicates the non-CD group and the cross indicates the CD group. (A) PhNR amplitude. (B) PhNR/b-wave amplitude ratio. (C) PhNR implicit time. CD, choroidal detachment; PhNR, photopic negative response.

The changes in ERG parameters preoperatively and postoperatively for each group are summarised in table 2. The scatter plots in figure 4 show the changes in the PhNR implicit time and amplitude and the PhNR/b-wave amplitude ratio.

**Table 2:** Comparison of full-field ERG parameters before and after the operation

	Non-CD group			CD group		
	Preoperatively	Postoperatively	P value	Preoperatively	Postoperatively	P value
Amplitude, mean (95% CI), µV						
a-wave	−4.4 (−5.0 to −3.8)	−4.9 (−5.4 to −4.3)	0.090	−4.9 (−6.0 to −3.8)	−3.1 (−4.2 to −2.0)	0.001*
b-wave	20.2 (18.1 to 22.3)	21.1 (18.7 to 23.4)	0.307	19.1 (14.5 to 23.8)	13.3 (9.3 to 17.3)	0.001*
PhNR	17.3 (15.6 to 19.1)	18.7 (16.7 to 20.6)	0.008*	17.0 (12.4 to 21.5)	11.4 (7.7 to 15.0)	0.002*
PhNR/b-wave amplitude ratio, mean (95% CI)	0.86 (0.84 to 0.89)	0.90 (0.87 to 0.93)	0.002*	0.88 (0.83 to 0.93)	0.85 (0.79 to 0.91)	0.219
Implicit time, mean (95% CI), ms						
a-wave	14.8 (14.4 to 15.2)	14.3 (14.0 to 14.7)	0.027*	15.3 (13.5 to 17.0)	15.0 (13.6 to 16.4)	0.811
b-wave	32.2 (31.5 to 32.9)	31.4 (30.9 to 32.0)	0.004*	33.0 (31.0 to 35.0)	33.7 (31.4 to 36.0)	0.300
PhNR	75.3 (72.6 to 78.0)	72.3 (70.4 to 74.3)	0.039*	75.2 (71.2 to 79.2)	79.2 (73.2 to 85.2)	0.120

The preoperative and postoperative full-field ERG parameters were compared using paired t-tests in both groups.

*P value <0.05 was considered significant

CD, choroidal detachment; ERG, electroretinographic; PhNR, photopic negative response.

In the non-CD group, the PhNR amplitude, PhNR/b-wave amplitude ratio and PhNR implicit time significantly improved after surgery. The PhNR amplitude changed from mean (95% CI) 17.3 (15.6 to 19.1) µV to 18.7 (16.7 to 20.6) µV (p=0.008). The PhNR/b-wave amplitude ratio changed from 0.86 (0.84 to 0.89) to 0.90 (0.87 to 0.93; p=0.002). The PhNR implicit time changed from 75.3 (72.6 to 78.0) to 72.3 (70.4 to 74.3) ms (p=0.039). In addition, the a-wave and b-wave implicit times significantly improved after surgery. The a-wave implicit time changed from 14.8 (14.4 to 15.2) ms to 14.3 (14.0 to 14.7) ms (p=0.027). The b-wave implicit time changed from 32.2 (31.5 to 32.9) ms to 31.4 (30.9 to 32.0) ms (p=0.004).

In the CD group, the PhNR amplitude significantly deteriorated after surgery. The PhNR amplitude changed from 17.0 (12.4 to 21.5) µV to 11.4 (7.7 to 15.0) µV (p=0.002). In addition, the a-wave and b-wave amplitudes significantly deteriorated after surgery. The a-wave amplitude time changed from −4.9 (−6.0 to −3.8) µV to −3.1 (−4.2 to −2.0) µV (p=0.001). The b-wave amplitude changed from 19.1 (14.5 to 23.8) µV to 13.3 (9.3 to 17.3) µV (p=0.001). The PhNR/b-wave amplitude ratio, PhNR implicit time, a-wave amplitude, and b-wave amplitude did not change significantly.

**Figure 5:**
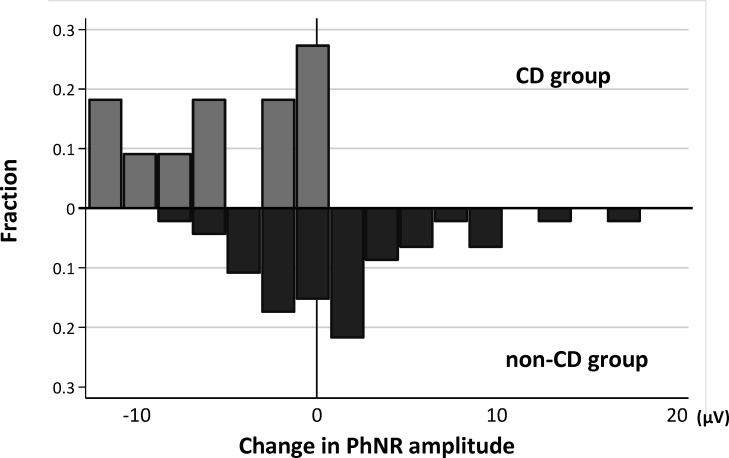
Distribution of change in the PhNR amplitude in the CD group and the non-CD group. CD, choroidal detachment; PhNR, photopic negative response.

Figure 5 shows the distribution of change in the PhNR amplitude in the CD group and the non-CD group. The postoperative change in PhNR amplitude was significantly lower in the CD group than in the non-CD group (p<0.001). Table 3 shows the results from the univariate and multivariate models investigating the relationship between the change in the PhNR amplitude and related factors. Postoperative IOP (p=0.031) and postoperative CD (p<0.001) were significantly associated with change in the PhNR amplitude in the univariate models. We separately examined the presence of postoperative CD and postoperative CD gradings with two different multivariable models. In the multivariate analysis, the presence of postoperative CD, CD grading 1 (p=0.048) and 3 (p=0.004) were significantly correlated with change in the PhNR amplitude.

**Table 3:** Association between change in PhNR amplitude and ocular variables: univariate and multivariable analysis

Variables	Change in PhNR amplitude, µV					
	Univariate model		Multivariate model 1		Multivariate model 2	
	Coefficients	P value	Coefficients	P value	Coefficients	P value
	(95% CI)		(95% CI)		(95% CI)	
Age (years) per 1 year.	0.00 (−0.12 to 0.13)	0.986	0.02 (−0.09 to 0.14)	0.712	0.01 (−0.11 to 0.13)	0.825
Gender (male/female)	−0.34 (−3.37 to 2.69)	0.823				
CCT (μm) per 1 µm	0.04 (−0.01 to 0.09)	0.157				
Axial length (mm) per 1 mm	−0.25 (−1.09 to 0.58)	0.544				
Preoperative BCVA, log MAR, per one unit	−2.67 (−7.62 to 2.28)	0.284				
Preoperative IOP per 1 mm Hg	0.09 (−0.14 to 0.33)	0.429				
Preoperative MD value, per 1 dB	−0.10 (−0.35 to 0.16)	0.449				
Preoperative RNFL thickness, per 1 µm	−0.08 (−0.19 to 0.03)	0.163				
Type of glaucoma surgery (trabeculectomy/ex-PRESS)	−0.67 (−3.64 to 2.30)	0.652				
Postoperative BCVA, log MAR, per one unit	−1.32 (-6.13 to 3.49)	0.584				
Change in BCVA, log MAR, per one unit	2.53 (−4.54 to 9.59)	0.477				
Postoperative IOP, per 1 mm Hg	0.43 (0.04 to 0.83)	0.031	0.17 (−0.23 to 0.57)	0.403	0.17 (−0.24 to 0.58)	0.408
Change in IOP, per 1 mm Hg	−0.04 (−0.25 to 0.17)	0.698				
Postoperative CD (yes/no)	−6.88 (−10.13 to –3.63)	＜0.001	−6.41 (−10.03 to –2.80)	0.001		
Postoperative CD (reference: no)						
Grade 1	−7.64 (−14.67 to –0.60)	0.034			−7.26 (−14.43 to –0.08)	0.048
Grade 2	−5.04 (−9.62 to –0.45)	0.032			−4.60 (−9.62 to 0.42)	0.072
Grade 3	−8.80 (−13.88 to –3.72)	0.001			−8.15 (−13.63 to –2.67)	0.004
Past cataract surgery (yes/no)	−0.07 (−3.48 to 3.33)	0.965				
Past glaucoma surgery (yes/no)	2.23 (−2.96 to 7.41)	0.393				
Past vitrectomy surgery (yes/no)	6.17 (−1.69 to 14.02)	0.121				
Past cerebrovascular event (yes/no)	1.35 (−5.26 to 7.95)	0.685				
Self-reported hypertension (yes/no)	−1.67 (−4.77 to 1.43)	0.286				
Self-reported diabetes (yes/no)	2.17 (−1.41 to 5.74)	0.229				

BCVA, best-corrected visual acuity; CCT, central corneal thickness; CD, choroidal detachment; IOP, intraocular pressure; logMAR, logarithm of the minimum angle of resolution; MD, mean deviation; PhNR, photopic negative response; RNFL, retinal nerve fibre layer.

### Intersession reproducibility

The CV values were 12.4% (95% CI 7.5% to 17.4%) for the PhNR amplitude, 2.4% (95% CI 1.1% to 3.7%) for PhNR/b-wave amplitude ratio and 6.0% (95% CI2.6% to 9.5%) for PhNR implicit time.

## Discussion

In this study, we demonstrated the rapid improvement in RGC function within several days after glaucoma filtration surgery by measuring PhNR using skin electrodes in the same eye preoperatively and postoperatively. The PhNR amplitude worsened after glaucoma surgery in patients with CD because of overfiltration.

Interestingly, the a-wave, b-wave and PhNR improved after glaucoma filtration surgery. This suggests the possibility that the reduction in IOP may be related to changes in blood flow in deeper layers. Deep macular microvasculature alteration in glaucomatous eyes has recently been reported.^11 21^ Further studies on whether this deep circulatory impairment can be improved by lowering IOP would provide an answer. Using OCTA, we recently reported microcirculatory disturbances in the macula before and after glaucoma surgery.^22 23^ The foveal avascular zone area was significantly reduced at 3 months after surgery. We concluded that capillary circulation may improve to a level detectable with OCTA. IOP, microcirculation and physiological improvements in function are considered closely related and act together quite early in the postoperative period.

RETeval is a relatively new ERG recording system that uses skin electrodes and is less invasive.^24^ RETeval PhNR is simple, reproducible and carries a low risk of infection when observing acute functional changes in the dense perioperative period.^24 25^ Using this method, it was possible to observe retinal function 2 hours after vitreous injection^26^ and several days after vitrectomy.^27^ In this study, we showed that skin electrode ERG can be used to evaluate retinal function in the early postoperative period, even in eyes after filtration surgery that cannot tolerate contact lenses and DTL electrodes.

Another notable finding of this study is the significant association between the presence of CD soon after postoperative glaucoma filtration surgery and changes in retinal function observed using skin electrode ERG. In some situations, mild CD is difficult to detect. Objective diagnosis of the presence or absence of CD in the early postoperative period is practical and could help decide on further management.^28^ This study showed that the behaviour of the ERG components recorded in the early postoperative period strongly correlated with the presence of CD. The a-wave, b-wave and PhNR waves deteriorated in the CD group. First, choroidal function may play a role. Miyake *et al* 15 analysed the electrooculogram of eyes with rhegmatogenous retinal detachment (RRD) and found that the preoperative values were significantly lower in eyes with CD than in those without. Choroidal dysfunction may affect outer retinal layer function, leading to changes in the a-wave and subsequently in the b-wave and PhNR. Second, another explanation is that the inner protrusion of the retinal surface caused by CD may have reduced the response of the ERG because of unequal stimulus light exposure. The fact that the amplitude deteriorated but the implicit time was relatively maintained is consistent with the latter explanation. Third, the effect of IOP on ERG changes should be considered. Miyake *et al* 29 used ERG to monitor retinal function during scleral buckling surgery in eyes with RRD. They observed a marked decrease in retinal function immediately after subretinal fluid drainage, but it improved with increased IOP caused by buckling. Therefore, the authors stated that the functional reduction was attributed to the effect of low IOP. In this study, eyes with CD also had a lower IOP than those without. However, rapid IOP reduction does not always cause reduction in the ERG response, as shown by studies of electrophysiological monitoring during intravitreal injection.^30 31^ Further studies are required to validate this mechanism.

Recently, Shin *et al* reported on CD grading using wide-angle photographs.^17^ The widespread use of wide-angle fundus photography has enabled objective interpretation of the degree of CD. In this study, we showed a significant association between CD grading and PhNR amplitude change. They showed the risk of CD was associated with pseudoexfoliation glaucoma, older age, and previous cataract surgery. Though there was no statistically significant difference between the two groups in this study, the results were consistent with the past reports by Shin as the CD group was older, had more cases of pseudoexfoliation glaucoma, systemic hypertension and diabetes, and previous cataract surgery.

### Limitations

The limitations of this study include its retrospective design, small sample size and lack of long-term postoperative data. It is possible that the deterioration of the PhNR amplitude may improve with the improvement of CD, and it is unclear how long the improvement of the PhNR amplitude will persist. Further studies with longer follow-up periods will clarify these associations. Second, it can be argued that the improved PhNR after surgery could be a result of improved media factors rather than the recovery of retinal function. Preoperative corneal oedema and combined cataract surgery may influence ERG quantitative measurements. In this study, the proportion of patients with a history past ocular surgery history was similar in the groups. Simultaneous cataract surgery was performed in 22 out of 46 eyes (47.8%) in the CD group and 3 out of 11 eyes (27.2%) in the non-CD group, and cataract surgery may have affected the postoperative changes in ERG. Tanikawa *et al* 32 conducted a detailed study on the effects of cataract surgery on ERG. They reported a significant increase in the a- and b-wave amplitudes, but not in PhNR, after cataract surgery. In addition, after excluding eyes with preoperative IOP higher than 30 mm Hg (five eyes) from the analysis, the results were similar (online supplemental table 1). Thus, the impact of media factors on PhNR, if any, may be considered inconsequential for the results of this study. Third, the influence of the hypotonic state on the ERG quality may be a cause of concern for selection bias. The comparisons of the ERG parameters between CD and non-CD groups should be interpreted with caution; however, the incidence of the excluded eyes due to insufficient ERG similar in the groups (data not shown).

10.1136/bjophthalmol-2021-320730.supp2 Supplementary data

## Conclusion

In conclusion, even in the early postoperative period within several days, the PhNR amplitude increased with IOP lowering following filtration surgery, and showed rapid functional recovery. However, the appearance of CD identified by wide-field fundus photography suggests that CD may arrest functional recovery, at least temporarily. The present results enhance understanding of the structural and functional recovery after glaucoma surgery and the role of postoperative CD.

## Data Availability

Data are available on reasonable request. The datasets generated and/or analysed during the current study are available from the corresponding author on reasonable request.
