# Supplementary figures and images for: Early changes in photopic negative response in eyes with glaucoma with and without choroidal detachment after filtration surgery
Source: Br J Ophthalmol. 2022 Apr 8;107(9):1295–302. doi: 10.1136/bjophthalmol-2021-320730 (PMC10447412; doi:10.1136/bjophthalmol-2021-320730)

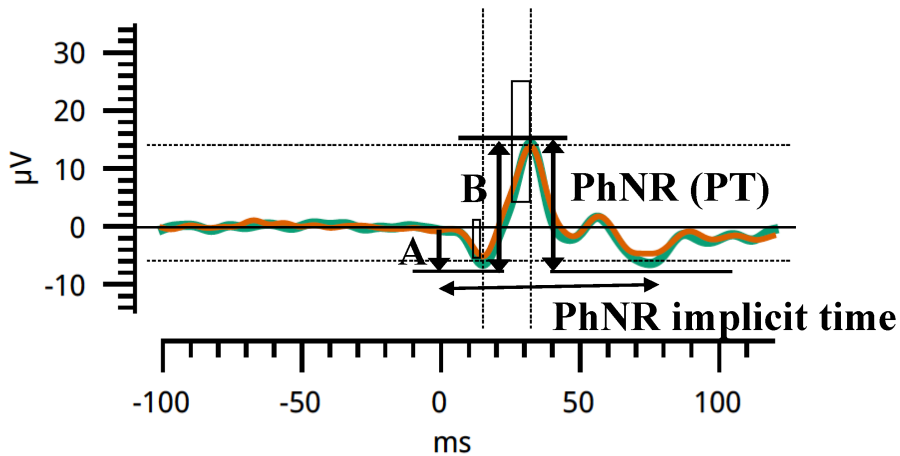

Supplement: Supplementary data [file bjophthalmol-2021-320730supp001.pdf]
