# Supplementary material for: Early changes in photopic negative response in eyes with glaucoma with and without choroidal detachment after filtration surgery
Source: Br J Ophthalmol. 2022 Apr 8;107(9):1295–302. doi: 10.1136/bjophthalmol-2021-320730 (PMC10447412; doi:10.1136/bjophthalmol-2021-320730)
Supplement: Supplementary data [file bjophthalmol-2021-320730supp002.pdf]

Supplemental Table 1. Association Between Change in PhNR amplitude and Ocular Variables: Univariate and Multivariable Analysis. (preoperative IOP < 30mmHg)

	Change in PhNR amplitude, μV					
	Univariate model		Multivariate model 1		Multivariate model 2	
Variables	**Coefficients (95% CI)**	***P value***	**Coefficients (95% CI)**	***P value***	**Coefficients (95% CI)**	***P value***
**Age (years) per 1 yr.**	0.06 (-0.10, 0.23)	0.448	0.08 (-0.06, 0.22)	0.284	0.07 (-0.08, 0.22)	0.349
**Gender (Male / Female)**	-0.85 (-4.08, 2.37)	0.598				
**CCT (μm) per 1 μm**	0.04 (-0.02, 0.09)	0.170				
**Axial Length (mm) per 1 mm**	-0.37 (-1.26, 0.53)	0.413				
**Preoperative BCVA, log MAR, per 1 unit**	-2.46 (-8.01, 3.08)	0.377				
**Preoperative IOP per 1mmHg**	0.12 (-0.23, 0.48)	0.500				
**Preoperative MD value, per 1 dB**	-0.12 (-0.42, 0.19)	0.440				
**Preoperative RNFL thickness, per 1 μm**	-0.07 (-0.20, 0.06)	0.288				
**Type of glaucoma surgery (Trabeculectomy / Ex-PRESS)**	-0.99 (-4.13, 2.16)	0.532				
**Postoperative BCVA, log MAR, per 1 unit**	-0.94 (-6.3, 4.41)	0.724				
**Change in BCVA, log MAR, per 1 unit**	2.4 (-4.8, 9.6)	0.506				
**Postoperative IOP, per 1mmHg**	0.64 (0.22, 1.06)	**0.003**	0.35 (-0.07, 0.76)	0.099	0.34 (-0.09, 0.77)	0.118
**Change in IOP, per 1mmHg**	-0.24 (-0.57, 0.09)	0.147				
**Postoperative CD (yes/no)**	-7.33 (-10.73, -3.92)	**<0.001**	-6.28 (-9.92, -2.63)	**0.001**		
**Postoperative CD (reference: no)**						
**Grade 1**	-7.61 (-14.72, -0.51)	**0.036**			-6.28 (-13.44, 0.87)	0.084
**Grade 2**	-5.73 (-10.86, -0.59)	**0.030**			-5.19 (-10.57, 0.20)	0.059
**Grade 3**	-8.78 (-13.92, -3.64)	**0.001**			-7.41 (-12.82, -2.00)	**0.008**
**Past cataract surgery (yes/no)**	0.04 (-3.48, 3.55)	0.984				
**Past glaucoma surgery (yes/no)**	2.36 (-2.94, 7.66)	0.375				
**Past vitrectomy surgery (yes/no)**	6.3 (-1.69, 14.28)	0.120				
**Past cerebrovascular event (yes/no)**	1.46 (-5.28, 8.2)	0.665				
**Self-Reported Hypertension (yes/no)**	-1.56 (-4.84, 1.71)	0.342				
**Self-Reported diabetes (yes/no)**	2.36 (-1.32, 6.03)	0.203				

Abbreviations:

PhNR, photopic negative response; CCT, central corneal thickness; BCVA, best-corrected visual acuity; IOP, intraocular pressure; MD, mean deviation; RNFL, retinal nerve fiber layer; CD, choroidal detachment
